# Pre‐Eclampsia and Its Associated Factors Among Pregnant Women in Ghana: A Retrospective Study

**DOI:** 10.1002/hsr2.72990

**Published:** 2026-08-05

**Authors:** Sabastian Adjei, Wilfred Sam‐Awortwi Jnr, Richmond Adu Boahen Boamah, Edward Osei Gyimah, William Kweku Payne, Harriet Yeboaa Diawuo, Samuel Kwame Sopuruchi Agomuo, Oscar Simon Olympio Mensah, Alfred Effah, Christian Obirikorang

**Affiliations:** ^1^ School of Anesthesia Komfo Anokye Teaching Hospital Kumasi Ghana; ^2^ Department of Molecular Medicine, School of Medical Sciences Kwame Nkrumah University of Science & Technology Kumasi Ghana

**Keywords:** maternal outcomes, neonatal outcomes, pre‐eclampsia, prevalence

## Abstract

**Background and Aims:**

Pre‐eclampsia remains a major cause of feto‐maternal death in Ghana, with a rising incidence linked to multiple factors that hinder prevention. Despite it's impact, few studies have explored associated risks. This study assessed the prevalence, risk factors, and maternal–neonatal outcomes associated with pre‐eclampsia among pregnant women at Kumasi South Hospital between January 2020 and May 2025.

**Methods:**

A retrospective descriptive study was conducted among 519 pregnant women who delivered at Kumasi South Hospital between January 2020 and May 2025. Data on sociodemographic, obstetric, clinical, and maternal–neonatal outcomes were extracted from medical records. Categorical variables were reported as frequencies and percentages, and binary logistic regression was used to identify independent predictors. *p*‐value < 0.05 was considered statistically significant.

**Results:**

The prevalence of pre‐eclampsia was 28.1%, with significant associations observed for adverse maternal/neonatal outcomes. After adjusting for multiple confounders, inadequate ANC visits [aOR = 4.79, 95% CI: 1.19–19.23, *p* = 0.027], headache or visual disturbances [aOR = 8.55, 95% CI: 1.90–38.46, *p* = 0.005], and low platelet count [aOR = 7.67, 95% CI: 1.96–30.02, *p* = 0.003] were associated with significantly increased odds of pre‐eclampsia. However, age [aOR= 0.41, 95% CI: 0.21–0.82, *p* = 0.012], and parity were also associated with reduced risk of pre‐eclampsia.

**Conclusion:**

Pre‐eclampsia was common among pregnant women delivering at KSH, negatively impacting maternal and neonatal outcomes. These findings highlight the need for closer antenatal monitoring and early identification of these risk factors to reduce the maternal and neonatal burden of pre‐eclampsia.

**Abbreviations**
ANC: antenatal care
aOR: adjusted odds ratio
APGAR: appearance, pulse, grimace, activity, respiration
BP: blood pressure
CI: confidence interval
cOR: crude odds ratio
HBV: hepatitis B virus
HELLP: hemolysis, elevated liver enzymes, low platelets
HIV: human immunodeficiency virus
IPTp‐SP: intermittent preventive treatment in pregnancy with sulfadoxine–pyrimethamine
KSH: Kumasi South Hospital
MCH: maternal and child health
NICU: neonatal intensive care unit
PE: pre‐eclampsia
SHS: Senior High School
SPSS: Statistical Package for Social Sciences

## 1 Introduction

Pre‐eclampsia is a leading cause of maternal morbidity and mortality worldwide, posing serious risks to both mothers and infants [1]. It is a hypertensive disorder of pregnancy, defined as new‐onset hypertension (systolic blood pressure ≥ 140 mmHg and/or diastolic blood pressure ≥ 90 mmHg) occurring after 20 weeks of gestation in a previously normotensive woman [2]. It may present with or without proteinuria and can cause end‐organ dysfunction, including liver dysfunction, thrombocytopenia, renal impairment, pulmonary edema, and neurological disturbances [2]. These complications can lead to severe health consequences for both maternal and neonatal outcomes, long‐term disability, and death [3]. Beyond the index pregnancy, women with a history of pre‐eclampsia carry an elevated lifetime risk of cardiovascular disease and type 2 diabetes later in life [4].

Pre‐eclampsia affects between 3% and 8% of pregnant women and accounts for 16% of all maternal deaths worldwide [5]. The burden is disproportionately higher in low‐ and middle‐income countries, where access to quality maternal healthcare remains limited [6]. Sub‐Saharan Africa accounts for the highest proportion of maternal deaths, with pre‐eclampsia contributing significantly to these losses [7]. The reported incidence rates in resource‐limited environments vary from 1.8% to 16.6% [8]. In Ghana, the prevalence has risen over time, from 4.6% in 2018 to 6.6% in 2020, with current estimates of 6.55%–7.3% of pregnancies being affected [9]. This increased burden in Ghana is due to maternal factors such as age, parity, multiple gestations, anemia, infections, low socioeconomic status, and limited educational [9, 10]. Also, poor antenatal attendance contributes this burden, despite Ghana's Free Maternal Health Policy [11].

Beyond its epidemiological burden, pre‐eclampsia substantially worsens neonatal outcomes through placental insufficiency and a compromised intrauterine environment. Neonates born to women with pre‐eclampsia are at increased risk of low birth weight, preterm delivery, and admission to the neonatal intensive care unit. Severe cases can result in intrauterine fetal death, perinatal asphyxia, and complications such as HELLP syndrome in mothers, further affecting neonatal survival.

Although pre‐eclampsia continues to pose a significant and growing health challenge in Ghana, comprehensive data on its prevalence, fetal and maternal outcomes remain limited, especially at the facility level. To address this gap, the current study investigated the prevalence, risk factors, and maternal–neonatal outcomes associated with pre‐eclampsia among pregnant women who delivered at the Kumasi South Hospital from January 2020 to May 2025.

## 2 Materials and Methods

### 2.1 Study Design and Setting

This retrospective cross‐sectional study was conducted at the Antenatal Care (ANC) clinic of Kumasi South Hospital, Ghana. Kumasi South Hospital is the second largest hospital in the southern part of Ashanti regional healthcare facilities. It is located within the Kumasi metropolis, with a population of over 500,000, and provides services, including general medical care, public health, diagnostics, training of medical residents, and specialist services, including Antenatal Care. The Maternal and Child Health (MCH) unit offers antenatal care, skilled delivery services, postnatal care, family planning, and child welfare services, handling approximately 1500 deliveries annually [12].

### 2.2 Study Population

The study included medical records of pregnant women who delivered at the Kumasi South Hospital, Ghana, between January 2020 and May 2025.

### 2.3 Inclusion and Exclusion Criteria

Medical records of pregnant women with singleton pregnancy delivery, containing complete sociodemographic and clinical data, and maternal–neonatal birth outcomes during the study period, were included. Patients with incomplete medical records, had multiple gestation pregnancies, or were referred during labor without antenatal records were excluded from the study.

### 2.4 Sample Size Estimation

The sample size for the study was obtained using Cochran's formula.

$$n=\frac{Z^{2}p\left(1-p\right)}{e^{2}},$$

where: *Z* is the standard normal variate at a confidence interval of 95% = 1.96. *p* is the estimated prevalence of pre‐eclampsia (0.088) in Ghana [13], and *e* is the margin of error = 0.05.

$$n\;(\text{Minimum number of participants})=\frac{1.96^{2}(0.088)\left(1-0.088\right)}{0.05^{2}}\;=\;123.28$$

Hence, a minimum of 124 participants is required for the study. To increase statistical power, a total of 519 women were included in the study.

### 2.5 Data Collection

Data were extracted from patients' medical records using a well‐structured questionnaire, complemented by information from the maternal birth register. Sociodemographic variables, including age, religion, occupation, and educational level were extracted by trained research assistants. Obstetric variables, including parity, gravidity, gestational age at childbirth, maternal pre‐pregnancy height and weight, prior pre‐eclampsia in previous pregnancies, history of stillbirth, family history of hypertension, readmission status, and maternal medical history were also obtained from the medical records. Clinical variables retrieved from medical records comprised renal function (proteinuria), liver function (liver enzymes), thrombocytopenia, pulmonary edema, anemia, chronic hypertension, diabetes mellitus, hepatitis B infection, prior pre‑eclampsia, and HIV serostatus. To prevent bias, missing data were excluded, as their inclusion could compromise the study outcome.

### 2.6 Data Quality Assurance

To ensure data quality, the data extraction form was pretested on a subset of records before data collection and revised for clarity based on the pretest. Research assistants were trained on the study's operational definitions and the extraction form prior to data collection. A random sample of extracted records was independently cross‐checked by a second reviewer to verify accuracy. All extracted data were reviewed for completeness and internal consistency, and any discrepancies were resolved by referring back to the original medical record.

### 2.7 Operational Definitions

Pre‐eclampsia was defined as gestational hypertension (≥ 140 mmHg systolic/90 mmHg diastolic) occurring after 20 weeks of gestation, accompanied by the presence of proteinuria. Proteinuria was assessed using the urine dipstick method, with women with a protein level of 1+ classified as having proteinuria [14]. Pre‐eclampsia was first recognized by obstetricians and midwives during routine care through blood pressure measurements and urine protein tests. For this study, we reviewed records and classified participants as pre‐eclampsia cases if their documented blood pressure and proteinuria met the criteria above. The APGAR score was assessed at 1 and 5 min after birth to evaluate newborn's immediate physiological conditions. This assessment comprised the appearance, pulse, grimace, activity, and respiration each scored from 0 to 2, yielding a total score ranging from 0 to 10. Higher scores indicated better neonatal adaptation, with scores of 7–10 reflecting good physiological status, scores of 4–6 indicating moderate difficulty requiring supportive care, and scores of 0–3 implying severe distress necessitating immediate resuscitative intervention [15].

### 2.8 Statistical Analyses

The collected data were entered, coded, edited, and cleaned using Microsoft Excel 2019. All analyses were performed using the Statistical Package for Social Sciences (SPSS), version 26.0. Categorical data were presented as frequencies and percentages (%), whilst continuous variables were presented as mean ± standard deviation. Associations between categorical variables were assessed using the Pearson Chi‐square test or Fisher's exact test, where expected cell frequencies were below 5. Binary logistic regression analysis was employed to test for associations and the independent predictors of pre‐eclampsia. To assess the validity of the multivariable logistic regression model, several diagnostic checks were performed. Multicollinearity among predictor variables was assessed using the Variance Inflation Factor (VIF), with VIF values below 5 considered acceptable. Model fit was evaluated using the Hosmer–Lemeshow goodness‐of‐fit test, and model performance was further assessed using the Nagelkerke *R* ^2^. Given the relatively small number of events observed for some predictor categories, the events‐per‐variable ratio was also considered when interpreting the stability of individual odds ratio estimates. Results of these diagnostic checks (VIF, Hosmer–Lemeshow test, and Nagelkerke *R* ^2^) are reported alongside the corresponding multivariable models in the Results section. All statistical tests were two‐sided, and a priori significance level of *p*‐values less than 0.05 was used throughout the analyses.

## 3 Results

### 3.1 Sociodemographic Characteristics of the Pregnant Women in the Study

Of the 519 pregnant women recruited in this study, more than half were between 25 and 35 years old (56.5%) and were unemployed (54.5%). Most pregnant women were married (49.7%). Approximately 44% had attained secondary education (43.9%) and had no history of birth (42.2%) (Table 1).

**TABLE 1:** Characteristics of the study participants.

Variable	Frequency (*n* = 519)	Percentage (%)
Age group (years)		
< 25	171	32.9
25–35	293	56.5
> 35	55	10.6
Marital status		
Married	258	49.7
Widowed	43	8.3
Single	218	42.0
Educational background		
No education	42	8.1
Basic	177	34.1
SHS	228	43.9
Tertiary	72	13.9
Employment status		
Employed	236	45.5
Unemployed	283	54.5
Parity		
Nulliparous	218	42.0
Primiparous	191	36.8
Multiparous	110	21.2

*Note:* Categorical data is presented as frequency and percentages.

Abbreviation: SHS, Senior High School.

### 3.2 Clinical and Obstetric Characteristics of Study Participants

The mean systolic and diastolic blood pressures were 121 ± 18.9 and 77.4 ± 16.2 mmHg, respectively. Majority had at least visited ANC 4 times and received IPTp‐SP during pregnancy (96.0%). Also, few participants had headaches or visual disturbances (17.5%), low platelet count (17.1%), presence of proteinuria (29.3%), pulmonary edema (24.5%), chronic hypertension (13.9%), diabetes mellitus (6.7%), and history of pre‐eclampsia (7.5%). Regarding infection, less than one‐tenth of participants had Hepatitis B (2.1%) and HIV (7.7%) (Table 2).

**TABLE 2:** Clinical and obstetric characteristics of the study participants.

Variable	Frequency (*n* = 519)	Percentage (%)
Systolic BP (mmHg)	121.3 ± 18.9	
Diastolic BP (mmHg)	77.4 ± 16.2	
ANC visits		
< 4 visits	59	11.4
≥ 4 visits	460	88.6
Proteinuria (yes)	152	29.3
Elevated liver enzymes (yes)	42	8.1
Headache or visual disturbances (yes)	91	17.5
Low platelet count (yes)	89	17.1
Pulmonary edema (yes)	126	24.3
Chronic hypertension (yes)	72	13.9
Diabetes mellitus (yes)	35	6.7
Hepatitis B (yes)	11	2.1
History of pre‐eclampsia (yes)	39	7.5
HIV status (yes)	40	7.7
IPTp‐SP received (yes)	498	96

*Note:* Categorical variables were presented as frequencies and percentages (%), whilst continuous variables were presented as mean ± standard deviation.

Abbreviations: ANC, antenatal care; BP, blood pressure; HIV, human immunodeficiency virus; IPTp‐SP, intermittent preventive treatment with sulfadoxine–pyrimethamine.

### 3.3 Prevalence of Pre‐Eclampsia Among Study Participants

**FIGURE 1:**
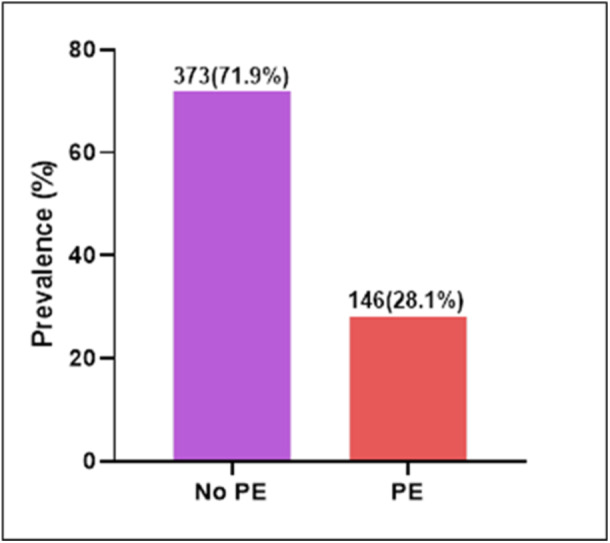
Prevalence of pre‐eclampsia among study participants. PE, pre‐eclampsia.

In this study, the prevalence of pre‐eclampsia was 28.1% (146/519), whilst the remaining 71.9% (373/519) had no pre‐eclampsia (Figure 1).

### 3.4 Association Between Neonatal/Maternal Outcomes and Pre‐Eclampsia

APGAR scores (*p* < 0.001), admission to the neonatal intensive care unit (*p* < 0.001), intrauterine fetal death (*p* < 0.001), low birth weight (*p* < 0.001), fetal growth restriction (*p* < 0.001), labor complications (*p* < 0.001), and HELLP syndrome (*p* < 0.001) were all significantly associated with pre‐eclampsia (Table 3).

**TABLE 3:** Association between neonatal/maternal outcomes and pre‐eclampsia.

		Pre‐eclampsia		
Variable	Total (*n* = 519)	No (*n* = 373)	Yes (*n* = 146)	*p*‐value
First‐minute APGAR score				**< 0.001**
Low (0–3)	45 (8.7)	7 (1.9)	38 (26.0)	
Intermediate (4–6)	205 (39.5)	106 (28.4)	99 (67.8)	
Normal (7–10)	269 (61.8)	260 (69.7)	9 (6.2)	
Fifth‐minute APGAR score				**< 0.001**
Low (0–3)	25 (4.8)	7 (1.9)	18 (12.3)	
Intermediate (4–6)	108 (20.6)	31 (8.3)	77 (52.7)	
Normal (7–10)	386 (74.4)	335 (89.8)	51 (34.9)	
NICU admission				**< 0.001**
No	404 (77.8)	355 (95.2)	49 (33.6)	
Yes	115 (22.2)	18 (4.8)	97 (66.4)	
Intrauterine fetal death				**< 0.001**
No	506 (97.5)	370 (99.2)	136 (93.2)	
Yes	13 (2.5)	3 (0.8)	10 (6.8)	
Birth weight				**< 0.001**
Low (< 2.5 kg)	178 (34.3)	51 (13.7)	127 (87.0)	
Macrosomia (> 4.0 kg)	11 (2.1)	10 (2.7)	1 (0.7)	
Normal (2.5–4.0 kg)	330 (63.6)	312 (83.6)	18 (12.3)	
Fetal growth restriction				**< 0.001**
No	448 (86.3)	369 (98.9)	79 (54.1)	
Yes	71 (13.7)	4 (1.1)	67 (45.9)	
Labor complications				**< 0.001**
No	293 (56.5)	240 (64.3)	53 (36.3)	
Yes	226 (43.5)	133 (35.7)	93 (63.7)	
Specific complications during labor				**< 0.001**
Prolonged labor	98 (43.3)	78 (58.6)	20 (21.5)	
Obstructed labor	12 (5.3)	9 (6.8)	3 (3.2)	
Antepartum hemorrhage	56 (34.8)	28 (21.1)	28 (30.1)	
Postpartum hemorrhage	53 (23.5)	16 (12.0)	37 (39.8)	
Incomplete placenta delivery	7 (3.1)	2 (1.5)	5 (5.4)	
HELLP syndrome				**< 0.001**
No	496 (95.6)	371 (99.5)	125 (85.6)	
Yes	23 (4.4)	2 (0.5)	21 (14.4)	

*Note:* Categorical data is presented as frequency and percentages. Chi‐square/Fisher exact test is presented. *p* < 0.05 are bolded and were considered statistically significant.

Abbreviations: APGAR, appearance pulse grimace activity respiration; HELLP, hemolysis elevated liver enzymes low platelets; NICU, neonatal intensive care unit.

### 3.5 Sociodemographic Predictors of Pre‐Eclampsia Among the Study Participants

In the univariate analysis, age above 35 years [cOR = 6.07, 95% CI: 3.11–11.86, *p* < 0.001] and being unemployed [cOR = 1.50, 95% CI: 1.001–22.22, *p* < 0.001] were associated with higher odds of pre‐eclampsia. Conversely, age 25–35 years [cOR = 0.62, 95% CI: 0.39–0.98, *p* = 0.030], having secondary school education [cOR = 0.33, 95% CI: 0.18–0.60, *p* < 0.001], and being either nulliparous [cOR = 0.33, 95% CI: 0.20–0.53, *p* < 0.001] or primiparous [cOR = 0.28, 95% CI: 0.17–0.46, *p* < 0.001] were associated with lower odds of pre‐eclampsia.

In the multivariable analysis, being 25–35 years old [aOR = 0.41, 95% CI: 0.21–0.82, *p* = 0.012] being either nulliparous [aOR = 0.41, 95% CI: 0.20–0.82, *p* = 0.012] or primiparous [aOR = 0.51, 95% CI: 0.27–0.95, *p* = 0.035] were protectors of pre‐eclampsia (Table 4).

**TABLE 4:** Sociodemographic predictors of pre‐eclampsia among the study participants.

Variable	PE (*n* = 146)	cOR (95% CI)	*p*‐value	aOR (95% CI)	*p*‐value
Age group					
< 25 (ref)	49 (33.6)	1.00	—	1.00	—
25–35	58 (39.7)	0.62 (0.40–0.95)	**0.030**	0.41 (0.21–0.82)	**0.012**
> 35	39 (26.7)	6.07 (3.11–11.86)	**< 0.001**	2.24 (0.86–5.83)	0.097
Marital status					
Married	85 (58.2)	1.49 (0.99–2.23)	0.051	1.22 (0.64–2.34)	0.551
Widowed	7 (4.8)	0.59 (0.25–1.40)	0.233	0.72 (0.25–2.10)	0.724
Single (ref)	54 (37.0)	1.00	—	1.00	—
Educational level					
No education	18 (12.3)	1.41 (0.65–3.08)	0.388	0.86 (0.33–2.28)	0.767
Basic	69 (47.3)	1.20 (0.68–2.13)	0.53	0.92 (0.43–1.98)	0.827
SHS	34 (23.3)	0.33 (0.18–0.60)	**< 0.001**	0.33 (0.17–0.65)	**0.002**
Tertiary (ref)	25 (17.1)	1.00	—	1.00	—
Employment status					
Employed (ref)	56 (38.4)	1.00	—	1.00	—
Unemployed	90 (61.6)	1.50 (1.001–2.22)	**0.042**	1.03 (0.58–1.84)	0.918
Parity					
Nulliparous	52 (35.6)	0.33 (0.20–0.53)	**< 0.001**	0.41 (0.20–0.82)	**0.012**
Primiparous	40 (27.4)	0.28 (0.17–0.46)	**< 0.001**	0.51 (0.27–0.95)	**0.035**
Multiparous (ref)	54 (37.0)	1.00	**—**	1.00	—

*Note:* Logistic regression *p*‐values are presented; *p* < 0.05 and bolded are statistically significant.

Abbreviations: 95% CI, 95th confidence interval; aOR, adjusted odds ratios; cOR, crude odds ratios; SHS, Senior High School.

Regression diagnostics confirmed the adequacy of the multivariable model presented in Table 4. The Hosmer–Lemeshow goodness‐of‐fit test indicated an acceptable model fit (*χ* ^2^(8) = 6.41, *p* = 0.602), and the model explained approximately 23.0% of the variance in pre‐eclampsia status (Nagelkerke *R* ^2^ = 0.230; Cox & Snell *R* ^2^ = 0.160). No multicollinearity was detected among the predictor variables, with Variance Inflation Factor (VIF) values ranging from 1.10 to 4.20.

### 3.6 Clinical and Obstetric Predictors of Pre‐Eclampsia Among the Study Participants

In the univariate analysis, having elevated liver enzymes [cOR = 145.26, 95% CI: 19.75–1068.51, *p* < 0.001], headache or visual disturbances [cOR = 23.57, 95% CI: 12.98–42.81, *p* < 0.001], low platelet count [cOR = 33.57, 95% CI: 17.34–64.99, *p* < 0.001], pulmonary edema [cOR = 122.08, 95% CI: 59.48–250.59, *p* < 0.001], chronic hypertension [cOR = 170.86, 95% CI: 41.01–711.86, *p* < 0.001], diabetes mellitus [cOR = 24.87, 95% CI: 8.60–71.93, *p* < 0.001], hepatitis B infection [cOR = 27.35, 95% CI: 3.47–215.68, *p* = 0.002], history of pre‐eclampsia [cOR = 62.97, 95% CI: 14.94–265.45, *p* < 0.001], and HIV infection [cOR = 4.39, 95% CI: 2.26–8.54, *p* < 0.001] were associated with higher odds of pre‐eclampsia.

After adjusting for confounders, attending ANC less than 4 times [aOR= 4.79, 95% CI: 1.19‐19.23, *p* = 0.027], having elevated liver enzymes [aOR = 47.70, 95% CI: 2.56–887.44, *p* = 0.010], headache or visual disturbances [aOR = 8.55, 95% CI: 1.90–38.46, *p* = 0.005], low platelet count [aOR = 7.67, 95% CI: 1.96–30.02, *p* = 0.003], pulmonary edema [aOR = 191.77, 95% CI: 47.17–779.67, *p* < 0.001], chronic hypertension [aOR = 72.09, 95% CI: 4.27–1216.99, *p* = 0.003], history of pre‐eclampsia [aOR = 20.34, 95% CI: 1.04–399.48, *p* = 0.047], and receipt of IPTp‐SP [aOR = 24.20, 95% CI: 1.35–434.45, *p* = 0.031] remained significantly associated with increased odds of pre‐eclampsia (Table 5).

**TABLE 5:** Clinical and obstetric predictors of pre‐eclampsia among study participants.

Variable	PE (*n* = 146)	cOR (95% CI)	*p*‐value	aOR (95% CI)	*p*‐value
ANC visits					
< 4	22 (15.1)	1.61 (0.91–2.84)	0.099	4.79 (1.19–19.23)	**0.027**
≥ 4 (ref)	124 (84.9)	1.00	—	1.00	—
Elevated liver enzymes					
No (ref)	105 (71.9)	1.00	—	1.00	—
Yes	41 (28.1)	145.26 (19.75–1068.51)	**< 0.001**	47.70 (2.56–887.44)	**0.010**
Headache/visual disturbances					
No (ref)	71 (48.6)	1.00	—	1.00	—
Yes	75 (51.4)	23.57 (12.98–42.81)	**< 0.001**	8.55 (1.90–38.46)	**0.005**
Low platelet count					
No (ref)	69 (47.3)	1.00	—	1.00	—
Yes	77 (52.7)	33.57 (17.34–64.99)	**< 0.001**	7.67 (1.96–30.02)	**0.003**
Pulmonary edema					
No (ref)	31 (21.2)	1.00	—	1.00	—
Yes	115 (78.8)	122.08 (59.48–250.59)	**< 0.001**	191.77 (47.17–779.67)	**< 0.001**
Chronic hypertension					
No (ref)	76 (52.1)	1.00	—	1.00	—
Yes	70 (47.9)	170.86 (41.01–711.86)	**< 0.001**	72.09 (4.27–1216.99)	**0.003**
Diabetes mellitus					
No (ref)	115 (78.8)	1.00	—	1.00	—
Yes	31 (21.2)	24.87 (8.60–71.93)	**< 0.001**	0.58 (0.03–13.27)	0.733
HBV					
No (ref)	136 (93.2)	1.00	—	1.00	—
Yes	10 (6.8)	27.35 (3.47–215.68)	**0.002**	0.01 (0.00–1.65)	0.074
History of PE					
No (ref)	109 (74.7)	1.00	—	1.00	—
Yes	37 (25.3)	62.97 (14.94–265.45)	**< 0.001**	20.34 (1.04–399.48)	**0.047**
HIV					
No (ref)	122 (83.6)	1.00	—	1.00	—
Yes	24 (16.4)	4.39 (2.26–8.54)	**< 0.001**	3.22 (0.56–18.45)	0.189
IPTp‐SP					
No (ref)	4 (2.7)	1.00	—	1.00	—
Yes	142 (97.3)	1.70 (0.56–5.13)	0.350	24.20 (1.35–434.45)	**0.031**

*Note:* Logistic regression *p*‐values are presented, *p* < 0.05 and bolded are statistically significant.

Abbreviations: 95% CI, 95th confidence interval; ANC, antenatal care; aOR, adjusted odds ratios; cOR, crude odds ratios; HBV, hepatitis B virus; HIV, human immunodeficiency virus; IPTp‐SP, intermittent preventive treatment in pregnancy; PE, pre‐eclampsia.

Regression diagnostics confirmed the adequacy of the multivariable model presented in Table 5. The Hosmer–Lemeshow goodness‐of‐fit test indicated an acceptable model fit (*χ* ^2^(4) = 2.59, *p* = 0.629), and the model explained approximately 88.3% of the variance in pre‐eclampsia status (Nagelkerke *R* ^2^ = 0.883; Cox & Snell *R* ^2^ = 0.614). No multicollinearity was detected among the predictor variables, with Variance Inflation Factor (VIF) values ranging from 1.02 to 1.94.

## 4 Discussion

Preeclampsia is a major hypertensive disorder of pregnancy and remains a leading cause of maternal and perinatal morbidity and mortality, particularly in resource‐limited settings. Its occurrence and severity are influenced by a range of sociodemographic, clinical, and obstetric factors, which may contribute to adverse pregnancy outcomes. In this study, we assessed the prevalence of preeclampsia and its independent predictors among women who delivered at Kumasi South Hospital between January 2020 and May 2025. We observed a high prevalence of preeclampsia in the study, with several factors independently associated with increasing odds of developing preeclampsia.

The prevalence observed in our study (28.1%) is relatively higher compared to other studies from Ghana and Ethiopia which reported 8.8% and 12.4%, respectively [13, 16]. The high prevalence likely results from Kumasi South Hospital's role in referring complex pregnancies from across the Ashanti Region, leading to a population with more severe and late‐presenting cases. Women presenting at referral facilities have often developed advanced hypertensive disease before first contact, inflating facility‐level prevalence beyond community‐based estimates. Similar elevated prevalence has been reported in studies from tertiary hospitals in Nigeria and Ethiopia, suggesting that facility type and case mix can influence observed outcomes [17, 18, 19]. Late presentations for antenatal care and delayed detection of hypertensive disorders may further contribute to the high prevalence observed [20]. The differences in prevalence could be attributed to varying study settings, methodological approaches, and seasonal factors. These findings highlight the persistent public health challenges posed by pre‐eclampsia.

Pre‐eclampsia was independently associated with adverse neonatal outcomes in this study. Neonates born to mothers with pre‐eclampsia had higher odds of low APGAR scores at 1 and 5 min, low birth weight, fetal growth restriction, NICU admission, and intrauterine fetal death. These effects indicate that the neonates were exposed to a compromised intrauterine environment even before delivery. The placenta was unable to sustain fetal growth and oxygenation throughout gestation. The progression from low APGAR scores and birth weight deficits to NICU admission and, in the most severe cases, intrauterine death. These findings are consistent with existing literature demonstrating that pre‐eclampsia leads to placental insufficiency, reduced uteroplacental perfusion, and chronic fetal hypoxia [19, 21, 22]. Similar associations have been reported in studies from Ghana and Ethiopia, emphasizing the significant neonatal burden attributable to maternal hypertensive disorder [23, 24]. Maternal outcomes were also significantly worse among women with pre‐eclampsia. The increased odds of complications, including prolonged labor, antepartum hemorrhage, postpartum hemorrhage, and HELLP syndrome, are consistent with findings from previous hospital‐based studies in sub‐Saharan Africa [25, 26]. The strong association between pre‐eclampsia and HELLP syndrome observed in this study aligns with the evidence that severe pre‐eclampsia may rapidly progress to life‐threatening maternal complications in the absence of early detection and timely intervention [27].

Maternal age was a significant predictor, with women in the optimal reproductive age group having lower odds of pre‐eclampsia compared to younger women. This finding is consistent with studies from Ghana, Kenya, and Tanzania that report increased risk among younger women, potentially due to biological immaturity, poor placentation, and limited health‐seeking experience [28, 29, 30]. Although advanced maternal age has also been linked with increased risk in other studies, the protective effects observed among women in the optimal age group in this study align with evidence that extremes of maternal age are associated with hypertensive disorders of pregnancy [31, 32].

Educational level also emerged as an independent predictor of pre‐eclampsia. Women with lower education level have higher chances of developing pre‐eclampsia, a finding consistent with previous studies in Ghana and Nigeria. Education is closely linked to socioeconomic status, health literacy, and access to healthcare services [33, 34, 35]. Women with higher level of education are more likely to recognize early warning signs, adhere to antenatal care schedules, and seek timely medical attention, thereby reducing the likelihood of severe disease.

Inadequate antenatal care attendance remained a significant predictor after adjustment, consistent with extensive literature demonstrating the protective role of regular antenatal visits. Studies across sub‐Saharan Africa have shown that women who attend fewer antenatal visits are at increased risk of undiagnosed or poorly managed hypertensive disorders [36, 37]. Multiparity was independently associated with higher odds of pre‐eclampsia in this study. Although primiparity is commonly reported as a risk factor, similar associations with multiparity have been documented in studies from Nigeria and Ethiopia [38, 39]. This may reflect cumulative vascular stress, short interpregnancy intervals, or the increasing prevalence of chronic conditions such as hypertension and obesity among multiparous women.

Clinical predictors demonstrated the strongest association with pre‐eclampsia. Chronic hypertension significantly increased the risk of pre‐eclampsia, aligning with well‐established evidence that women with pre‐existing hypertension are at high risk of developing superimposed pre‐eclampsia. This finding is consistent with studies from both high‐income and low‐income settings, which identify chronic hypertension as one of the strongest predictors of pre‐eclampsia [38, 40]. Additionally, abnormal clinical findings, including elevated liver enzymes, thrombocytopenia, headaches or visual disturbances, and pulmonary edema, remained significant predictors after adjustment. These findings are consistent with the multisystem pathophysiology of pre‐eclampsia and mirror results from studies conducted in Ghana and Ethiopia [41, 42, 43]. Pulmonary edema, which showed particularly high adjusted odds, has been widely reported as a marker of severe diseases, as it is often associated with delayed presentation and advanced maternal compromise. Interestingly, receipt of IPTp‐SP was also associated with increased odds of preeclampsia in our study. IPTp‐SP is primarily used to prevent malaria in pregnancy; its relationship with preeclampsia remains unclear. The near‐universal uptake of IPTp‐SP in our cohort (96.0%) also left a very small comparison group, which likely contributed to the instability of this estimate. A study from Nigeria reported a 3.73% prevalence of preeclampsia despite high IPTp‐SP coverage (73.9%), suggesting that while IPTp‐SP may reduce malaria‐related complications, but not directly prevent hypertensive disorders [44].

The strength of this study is its comprehensive approach, integrating independent predictors with maternal and neonatal outcomes of pre‐eclampsia in KSH. However, the study had some limitations. As a retrospective study, our analysis was limited by the data available in patients' records. Variability in data quality and missing data reduced the number of records that met our inclusion criteria, potentially limiting statistical power. Additionally, biochemical parameters such as proteinuria quantification, liver enzyme levels, and blood pressure were retrieved from existing medical records, and the accuracy of the results depended on the quality and completeness of the data sources. A prospective multisite cohort study with standardized data collection protocols, including laboratory confirmation, would provide more robust and actionable estimates of risk.

## 5 Conclusion

Approximately three in 10 of pregnant women suffer from preeclampsia in Ghana with factors such as maternal age, educational status. Parity, number of ANC visits, elevated liver enzymes, headache or visual disturbance, low platelet count, pulmonary edema, chronic hypertension, history of preeclampsia, and IPTp‐SP administration being independent predictors of preeclampsia. Healthcare providers should reinforce education on regular antenatal visits, raise community awareness of pregnancy danger signs, incorporate pre‐conception hypertension screening into reproductive health services, and establish standardized protocols for preeclampsia management and postpartum follow‐up to reduce its impact and complications.

## Author Contributions

**Sabastian Adjei:** methodology, investigation, writing – original draft, writing – review and editing. **Wilfred Sam‐Awortwi Jnr:** conceptualization, writing – original draft, writing – review and editing. **Richmond Adu Boahen Boamah:** conceptualization, writing – original draft, writing – review and editing. **Edward Osei Gyimah:** writing – original draft, writing – review and editing, methodology, formal analysis. **William Kweku Payne:** methodology, writing – original draft, writing – review and editing. **Harriet Yeboaa Diawuo:** writing – original draft, writing – review and editing, methodology. **Samuel Kwame Sopuruchi Agomuo:** writing – review and editing, writing – original draft, formal analysis. **Oscar Simon Olympio Mensah:** writing – original draft, writing – review and editing, formal analysis. **Alfred Effah:** formal analysis, writing – review and editing, writing – original draft. **Christian Obirikorang:** conceptualization, supervision, writing – review and editing, writing – original draft, formal analysis.

## Funding

The authors have nothing to report.

## Disclosure

The lead author, Christian Obirikorang, affirms that this manuscript is an honest, accurate, and transparent account of the study being reported; that no important aspects of the study have been omitted; and that any discrepancies from the study as planned (and, if relevant, registered) have been explained.

## Ethics Statement

Ethical approval for the study was obtained from the School of Anesthesia‐Kumasi (MOH/SOAK/08‐25/78/23) and the ethics committee of the Kumasi South Hospital prior to conducting the research. All patient records were handled anonymously, with strict confidentiality maintained throughout data extraction, storage, and analysis, in accordance with the ethical principles outlined in the Declaration of Helsinki.

## Consent

The authors have nothing to report.

## Conflicts of Interest

The authors declare no conflicts of interest.

## Data Availability

The data that support the findings of this study are available from the corresponding author upon reasonable request.
